# UTILIZATION OF THE LONG COVID DIAGNOSIS CODE IN US NURSING HOME RESIDENTS

**DOI:** 10.1093/geroni/igae098.2344

**Published:** 2024-12-31

**Authors:** Yasin Abul, Daniel Harris, Preeti Chachlani, Kaley Hayes, Vincent Mor, Stefan Gravenstein

**Affiliations:** Brown University, Providence, Rhode Island, United States; Brown University, Providence, Rhode Island, United States; Brown University, Providence, Rhode Island, United States; Brown University, Providence, Rhode Island, United States; Brown University, Providence, Rhode Island, United States; Brown University, Providence, Rhode Island, United States

## Abstract

The National Center for Health Statistics implemented the ICD-10 code U09.9 for “Long COVID” or “post COVID-19 condition” on October 1st, 2021. Overall utilization, trends, and variation in the use of the U09.9 ICD code in long term care (LTC) residents remains understudied. We therefore conducted a serial cross sectional study using the COVVAXAGE database, by linking CVS Health and Walgreens’ customers data to the 100% Medicare enrollment files. Monthly, from October 2021 through April 2023, we identified individuals living in LTC and enrolled continuously in Medicare FFS for 12 months. We used Medicare Part A and B claims to identify long COVID (U09.9). We estimated the monthly occurrence of a long COVID code among all LTC residents and stratified by a recent (8 weeks) and latent (52 weeks) diagnosis of COVID-19, and by region. We identified over 200,000 LTC residents each month (mean age = 82.8; 69% women; 79.4% White; 11.9% Black). The overall long COVID coding rate was lowest in April, 2023 (1 per 10,000 residents) and highest in February, 2022 (14 per 10,000 residents). Residents with recent COVID-19 diagnosis had the highest long COVID coding rate (e.g., 58 per 10,000 residents) in January 2023. Temporal trends in long COVID coding rates varied widely by geography and were cyclical, following epidemic trends in LTC residents. Long COVID U09.9 ICD code documentation rates were generally low and varied in LTC residents. Remarkable variation in coding rates by region, urbanicity, and frailty status occurred, and needs further study.

